# Mucocutaneous Manifestations and Associated Factors in Patients with Crohn’s Disease

**DOI:** 10.5152/tjg.2022.21750

**Published:** 2022-11-01

**Authors:** Yusuf Kayar, Ramazan Dertli, Şevki Konür, Mehmet Ağın, Abdullah Al Kafee, Bülent Baran, Aslı Çiftçibaşı Örmeci, Filiz Akyüz, Kadir Demir, Fatih Beşışık, Sabahattin Kaymakoğlu

**Affiliations:** 1 Division of Gastroenterology, Department of Internal Medicine, Van Teaching and Research Hospital, Health Sciences University, Van, Turkey; 2 Department of Internal Medicine, Van Teaching and Research Hospital, Health Sciences University, Van, Turkey; 3 Division of Gastroenterology, Department of Pediatrics, Van Teaching and Research Hospital, Health Sciences University, Van, Turkey; 4 Department of Biomedical Engineering, İstanbul University, İstanbul, Turkey; 5 Division of Gastroenterology, Department of Internal Medicine, Koç University, İstanbul, Turkey; 6 Division of Gastroenterology, Department of Internal Medicine, İstanbul University, İstanbul, Turkey

**Keywords:** Crohn’s disease, mucocutaneous involvement, risk factors

## Abstract

**Background:**

One-third of all extraintestinal manifestations are mucocutaneous findings in patients with Crohn’s disease and there is a relationship between some risk factors. Our aim is to evaluate factors associated with mucocutaneous manifestations in our cohort of patients with Crohn’s disease with a follow-up duration of up to 25 years.

**Methods:**

In the study, 336 patients with Crohn’s disease who were followed up between March 1986 and October 2011 were included**.** The demographic characteristics, Crohn’s disease-related data, and accompanying mucocutaneous manifestations were recorded. The cumulative probability of mucocutaneous extraintestinal manifestations and possible risk factors were analyzed.

**Results:**

Oral and skin involvement were detected in 109 (32%) and 31 (9.2%) patients, respectively. The cumulative probability of developing oral and skin manifestations were 43.2% and 20.3%, respectively. Cox regression analysis showed that female gender (odds ratio: 3.28, 95% CI: 1.51-7.14, *P *= .003) and corticosteroid use (odds ratio: 7.88, 95% CI: 1.07-57.97, *P *= .043) are independently associated with the development of skin manifestations, while family history (odds ratio: 3.59, 95% CI: 2.18-5.93, *P *< .001) and inflammatory-type disease (odds ratio: 1.776, 95% CI: 1.21-2.61, *P *= .004) were independently associated with the development of oral ulcers.

**Conclusion:**

Mucocutaneous extraintestinal manifestations are associated with female gender, corticosteroid use, family history, and disease type in a large cohort of patients with Crohn’s disease. Defining the specific relationships of immune-mediated diseases will help to better understand the pathogenesis of Crohn’s disease and associated mucocutaneous manifestations and to use more effective treatments.

**Main Points**

The cumulative probability of developing skin and oral involvement in their 25-year follow-up was 20.3% and 43.2%, respectively. Compared to the other population, skin involvement was similar, but oral involvement was more common in the Turkish population.
Cox regression analysis showed that female gender (OR: 3.28, 95% Cl: 1.51-7.14, *P* = .003) and corticosteroid use (OR: 7.88, 95% Cl: 1.07-57.97, *P* = .043) are independently associated with the development of skin manifestations, family history (OR: 3.59, 95% Cl: 2.18-5.93, *P* < .001) and inflammatory-type disease (OR: 1.776, 95% Cl: 1.21-2.61, *P* = .004) were independently associated with the development of oral ulcers.
Defining the specific relationships of immune-mediated diseases will help to better understand the pathogenesis of Crohn’s disease and associated mucocutaneous manifestations and to use more effective treatments.

## Introduction

Inflammatory bowel disease (IBD) is a group of diseases consisting of Crohn’s disease (CD) and ulcerative colitis, with chronic inflammation in the gastrointestinal tract. In genetically sensitive individuals, IBD is thought to develop secondary to inappropriate and persistent inflammatory responses following the complex interaction of genetic, environmental, and immunoregulatory factors with the gut microbiota and antigens. Chronic inflammation in the intestines causes edema, ulceration, bleeding, diarrhea, and fluid–electrolyte loss.^1,2^

Although CD is primarily defined as chronic inflammation of the intestines, it has been observed that it is not limited to the gastrointestinal tract but affects many organ systems.^3,4^ As a result of extraintestinal manifestations (EIMs) involving the musculoskeletal system, skin, eyes, hepatobiliary system, kidneys, lungs, cardiovascular, hematological, and immunological systems, it negatively affects the quality of life by impaired functional status.^5,6^ Extraintestinal manifestations are sometimes mild and transient, sometimes progressing very severely, and maybe more prominent than intestinal symptoms.^7^ Joint and mucocutaneous involvements are frequently seen in CD.^5,6^ It is reported that EIMs occur approximately in one-third of CD patients and one-third of these EIMs are mucocutaneous manifestations, including but not limited to erythema nodosum (EN), pyoderma gangrenosum (PG), and aphthous ulcers.^8-11^

It is known that some genetic and environmental factors play a role in the development of mucocutaneous manifestations in patients with CD. It was also reported that 1 EIM may trigger the development of another EIM and the probability of developing multiple EIMs ranged from 4% to 25%. This is thought to be due to the common underlying pathological pathways and genetic features.^10,11^ In studies examining the various risk factors associated with the development of mucocutaneous findings, the results appear to be contradictory, as patient populations and study designs are different. However, factors such as disease activity, gender, location, type of disease, family history, drugs used, and perianal disease are reported to be very important in skin and oral involvement.^12-14^ We aimed to investigate mucocutaneous involvement only in patients with CD, since skin involvement in ulcerative colitis patients is better known and oral involvement differs from other populations in our country. So in our study, we aimed to determine the frequency of oral aphthous stomatitis and skin involvement and the cumulative probability of the development of mucocutaneous manifestations at 25-year follow-up. In addition, we also aimed to identify independent risk factors that influence the development of oral aphthous stomatitis and skin involvement.

## Materials and Methods

### Study Design

In the study, 336 patients who were followed up between March 1986 and October 2011 with the diagnosis of CD in the gastroenterology outpatient clinic of our hospital were included. Our study was designed as a single-center retrospective cohort study. The diagnosis of CD was made using radiological, endoscopic, histopathological, and clinical examinations. There is no single gold standard for the diagnosis of CD and diagnosis is made by clinical evaluation including a detailed history, physical examination, and combination of endoscopic findings, histology, radiologic findings, and laboratory investigations. The typical symptoms of CD are abdominal pain, diarrhea, and weight loss. Initial laboratory investigations include CBC, C-reactive protein, and serum chemistry. Ileocolonoscopy and biopsies are recommended as the first-line procedures to establish the diagnosis. Typical endoscopic findings of CD are a non-continuous distribution of longitudinal ulcers, cobblestone mucosal appearance, and aphthous ulcerations arranged in a longitudinal fashion. Focal and patchy chronic inflammation, focal crypt irregularity, and non-caseating granulomas are usual microscopic features of CD. Patients with CD who were followed up for at least 6 months were included in the study. Patients with ulcerative colitis, indeterminate colitis or unclassified IBD, and other inflammatory diseases of the bowel were not included in the study. The patients who were included in the study were confirmed after the systematic physical examination by a gastroenterologist and then evaluated by a dermatologist for the diagnosis of mucocutaneous lesions. Before starting the study, a power analysis was performed to determine the number of patients to be included in the study and the follow-up period.

### Mucocutaneous Manifestations

Patients with existing oral and skin symptoms were consulted with the dermatology department. The findings were accepted as an EIM of CD after exclusion of other primary or secondary etiologies that would lead to similar findings. Drug-related, malnutrition, and malabsorption-related pathologies were not accepted as EIM. The studies were analyzed, and the presence of psoriasis was sometimes evaluated as an accompanying autoimmune disease and sometimes as an EIM. We evaluated psoriasis as EIM. The time of development of oral and skin findings was recorded either it is before the diagnosis of CD or during the follow-up. The cumulative probability for the development of mucocutaneous findings was calculated.

*Erythema nodosum:* Erythema nodosum was defined as a rapid onset, bilateral, multiple, symmetrical and painful red nodule(s). It was observed that the lesions healed in approximately 3-5 weeks without leaving ulcers and scars. In biopsies taken by the dermatologist, immune complex deposition was observed in the subcutaneous adipose tissue.

*Pyoderma gangrenosum: *Pyoderma gangrenosum was defined as an inflammatory papule or pustule that can progress to a painful ulcer. Pyoderma gangrenosum often develops in the lower extremities, starts as an erythematous area, but later turns into a painful ulcer with sterile necrotic areas. Since the current findings may occur in syphilis, bacterial, fungal and mycobacterial infections, exposure to iodide and bromide, and spider bites, biopsy, and culture were taken from all patients, and the presence of other etiologies was excluded.

*Oral involvement: *Oral involvement was defined as aphthous ulcers or pain in the mouth and gums. Oral symptoms and findings were accepted as CD-linked EIMs after eliminating inflammatory or rheumatological diseases including infections, Reiter’s syndrome, and Behçet’s disease, which may cause similar clinical findings.

### Definitions and Classification of the Disease

Demographic data of patients, age of onset, disease duration, positive family history of IBD, location and type of disease, and presence of perianal disease were documented. Age of onset, location and type of disease, and presence of perianal disease were determined according to the Montreal classification.^15^ Type of disease was classified into 3 different categories including inflammatory, structuring, and penetrating-type considering the presence or development of intestinal complications such as abscess, fistula, and stricture.^16^ Patients with perianal fissure, abscess, and perianal fistula were determined as isolated perianal diseases based on the Montreal classification.^15^ Patients’ smoking status was questioned and all smokers were referred to the smoking cessation clinics. Surgery due to intestinal complications and/or perianal disease was recorded as a history of surgical operation. The relationship between the development of mucocutaneous findings and age of disease onset, location, type and duration of disease, gender, family history, perianal disease, surgery, and smoking were analyzed.

### Treatments

Medications including aminosalicylates, antibiotics, budesonide or systemic corticosteroids, immunosuppressive drugs (6-mercaptopurine, azathioprine, and methotrexate), and biologics (infliximab or adalimumab) were administered according to individualized treatment needs and indications. Treatment failures, perianal disease, and uncontrolled extraintestinal manifestations were treated by using biologic agents after January 2003. We used aminosalicylates in patients with mild forms of the disease. Systemic corticosteroids and budesonide were used as the main treatment for remission induction and flare-up episodes. Immunosuppressive drugs (azathioprine 2-2.5 mg/kg, 6-mercaptopurine 1-1.5 mg/kg) were administered for the maintenance of the remission, and intramuscular methotrexate (25 mg/week) was used as an alternative immunosuppressive in patients with intolerance to thiopurine therapy. Anti-TNF treatment was applied in patients whose disease activity continued despite standard immunosuppressive therapy. The relationship between the development of mucocutaneous manifestations and treatment was analyzed.

### Ethics Statement

All participants provided their written consent for participation in the study. Approval for conducting this study was obtained from the Local Ethical Committee (2011/831-577) at our hospital. All procedures were in accordance with the ethical standards of the committee on human experimentation of our institution and with the Declaration of Helsinki.

### Statistical Analysis

The results of our study were analyzed with the Statistical Package for Social Sciences 19.0 (IBM Corp.; Armonk, NY, USA) program. The data that had continuous values were given as (mean ± standard deviation), and the categorical data were given as frequency and percentage (n, %). The cumulative probabilities of developing extraintestinal manifestations during the follow-up were calculated using Kaplan–Meier method and log-rank test. The chi-square test was employed to test the categorical data. Logistic regression analysis was used to determine the risk factors affecting the development of mucocutaneous findings in Crohn’s patients and Cox regression analysis was used to determine the independent risk factors. The results are reported as hazard ratios (HR) with 95% CIs. In these analyzes, *P* < .05 value was considered statistically significant.

## Results

Three hundred thirty-six patients with CD were included in the study (2535 patient-years follow-up with an average of 7.54 years [range: 0.5-25.4 years]); 55.4% (n = 186) of the patients were male. While the number of patients with a disease duration <10 years was 238 (70.8%), 98 (29.2%) patients had a disease duration ≥10 years. The mean age at the time of diagnosis was 30.6 (range: 10.3-68.2) years.

### Mucocutaneous Findings Accompanying CD

Oral aphthous ulcer(s) and skin involvement were detected in 109 (32%) and 31 (9.2%) patients, respectively. The distribution of skin manifestations were as follows: EN in 12 (3.6%) patients, PG in 12 (3.6%), psoriasis in 2 (0.6%), dermatitis in 2 (0.6%), erythema multiforme in 1 (0.3%), and erythema bullosa in 1 (0.3%) patient(s). While 14 (45.1%) of those with skin involvement did present before the diagnosis of CD, 17 (54.9) presented after the diagnosis of CD. Forty-six (42.2%) patients with oral involvement had associated symptoms before the diagnosis of CD, and 63 (57.8%) patients were diagnosed during the course of CD.

### Cumulative Risk of Mucocutaneous Manifestations

**Figure 1.**
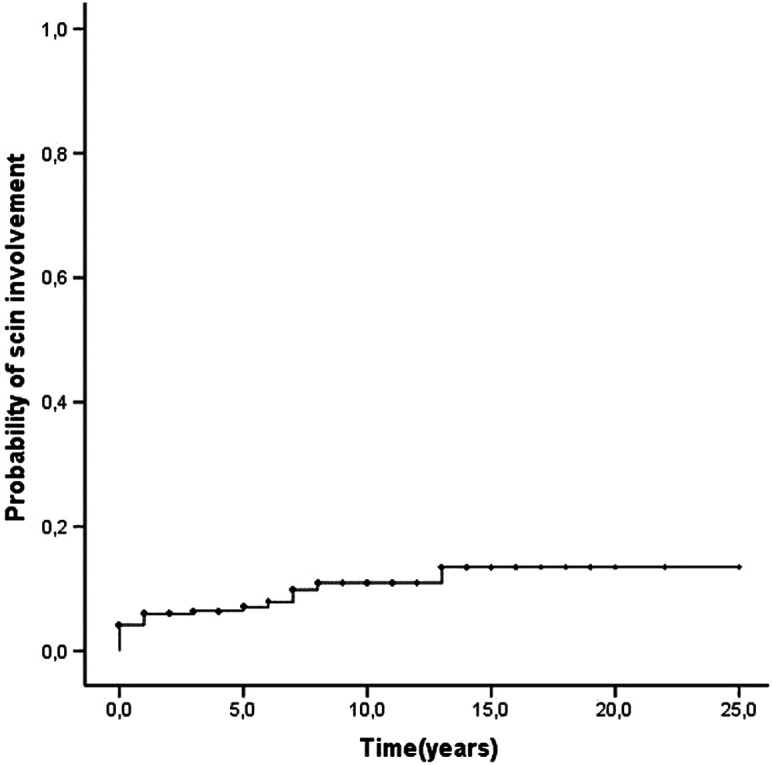
Cumulative probability of skin involvement in patients with CD. CD, Crohn’s disease.

**Figure 2.**
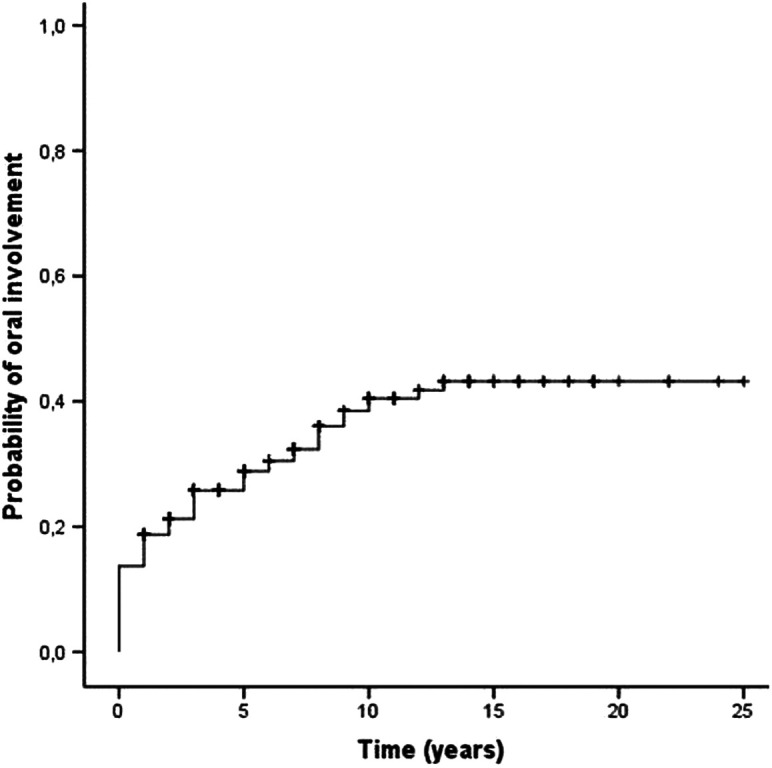
Cumulative probability of oral involvement in patients with CD. CD, Crohn’s disease.

The cumulative probability of development of mucocutaneous involvement during the follow-up was calculated. The cumulative probability of developing skin involvement was 4.2% of patients when diagnosed CD, 6.7% of patients in the first year of the disease, 8.3% of the 5th year of the disease, 13.6% of the 10th year of the disease, 20.3% of the 15th year of the disease, 20.3% of the 20th year of the disease, 20.3% of the 25th year of the disease (Figure 1). The cumulative probability of developing oral involvement was 13.7% of patients when diagnosed CD, in 18.8% of patients in the first year of the disease, 28.8% of the fifth year of the disease, 40.5% of the 10th year of the disease, 43.2% of the 15th year of the disease, 43.2% of the 20th year of the disease, and 43.2% of the 25th year of the disease (Figure 2).

### The Relationship Between the Development of Mucocutaneous Extraintestinal Findings and Risk Factors

Female gender, colon involvement, and perianal disease were significantly associated with the development of skin findings in the univariate analyses (*P* < .005). The disease duration, age of onset, disease type, smoking status, family history of IBD, and need for surgical operation were not found to be associated with the development of skin findings (Table 1). There was no relationship between the use of immunomodulators and the development of skin findings. However, the need for corticosteroids, antibiotics, and anti-TNFs was significantly associated with the development of skin findings (Table 2). It was observed that oral ulcers were detected significantly higher in patients with skin involvement (*P* < .001). When multivariate logistic regression analysis is performed to determine independent risk factors, female gender (HR: 3.38, 95% CI: 1.51-7.59, *P *= .003), presence of perianal disease (HR: 2.64, 95% CI: 1.25-5.58, *P *= .011), corticosteroid use (HR: 10.13 95% CI: 1.36-75.54, *P *= .024), antibiotic use (HR: 3.28, 95% CI: 1.23-8.78, *P *= .018), and anti-TNF use (HR: 2.31, 95% CI: 1.10-4.88, *P *= .028) were found to be independent risk factors for the development of skin findings.

Development of oral findings was found to be significantly higher in patients with female gender, colon involvement, positive family history, inflammatory-type CD, and those without a history of intestinal surgery (*P *< .05). Disease duration, age of onset, smoking status, and perianal disease were not found to be associated with the development of oral ulcers (Table 3). We did not find any relationship between the development of oral findings and the use of corticosteroids, antibiotics, immunomodulators, and anti-TNFs (Table 4). Skin involvement was found to be significantly higher in patients with oral involvement (*P *< .001). When multivariate logistic regression analysis is performed to determine independent risk factors; female gender (HR: 1.76, 95% CI: 1.11-2,80, *P *= .016), positive family history (HR: 9.37, 95% CI: 3.40-25.87, *P *< .001), inflammatory-type CD (HR: 2.01, 95% CI: 1.26-3.19, *P *= 0.003) and absence of a surgical operation history (HR: 1.67 95% CI: 1.04-2.67, *P *= .034) were found to be independent risk factors for the development of oral ulcers.

### Independent Risk Factors Associated with the Development of Mucocutaneous Involvement

Multivariate Cox regression analysis showed that female gender (HR: 3.28, 95% CI: 1.51-7.14, *P *= .003) and corticosteroid use (HR: 7.88, 95% CI: 1.07-57.97, *P *= .043) were found to be independent risk factors for the development of skin findings. Family history (HR: 3.59, 95% CI: 2.18-5.93, *P* < .001) and presence of inflammatory disease (HR: 1.78, 95% CI: 1.21-2.61, *P *= .004) were found to be independent risk factors for the development of oral ulcers (Table 5).

## Discussion

Crohn’s disease is known to be associated with extraintestinal findings. In studies with a large patient population, the rate of EIMs was reported to be over 40%.^11,17^ Although many organ systems may be involved, mucocutaneous involvement is the most common manifestation of IBD.^18^ The studies from various countries reported different results regarding the frequency of skin manifestations. Prevalence of EN and PG is above 15%, and the frequency of oral aphthae is reported between 0.7% and 33%.^12,19-21^ In the study including 1840 CD patients in the Swiss population, it was reported that mucocutaneous involvement was present in 354 patients.^22^ In another study including 118 CD patients in the Turkish population, 22 (16.9%) patients had skin involvement consisting of EN, pyoderma gangrenosum and psoriasis, and oral aphthous ulcers developed in 39 (33%) patients.^12^ In a study from Portugal, 44.4% of the patients had skin involvement among 342 patients with IBD, but this rate was 14.9% when nutritional deficiency and drug-related manifestations were excluded.^23^ Similarly, in a study from Greece including 1001 CD patients, 169 patients (16.9%) had mucocutaneous involvement of whom 69 (6.9%) had oral aphthous ulcers.^8^ In our study, skin involvement was observed in 9.2% of patients and oral aphthous ulcers developed in 32%. In addition, after up to 25 years of follow-up, the cumulative probability of skin involvement and oral aphthae development was 20.3% and 43.2%, respectively. Mucocutaneous involvement had developed in approximately 40% of patients before the diagnosis of CD and there was a significant correlation between the development of skin and oral manifestations.

In the published studies, the frequency of mucocutaneous findings in CD patients varies as well as the factors affecting EIM development. Roth et al.^22^ showed that mucocutaneous involvement was significantly higher in women, in those with a positive family history, in early disease onset and perianal abscess. In addition, it was shown that corticosteroid, immunomodulator, antibiotics, and anti-TNF drug usage were higher in those with mucocutaneous involvement.^22^ Vide et al.^23^ reported that mucocutaneous involvement was significantly higher in patients with an early-onset disease and in women. Many studies have reported that EN and PG development is higher in women and patients with colon involvement.^6,24,25^ Similarly, oral ulcer development is more common in patients with positive family history and colon involvement, but its relationship with gender is not clear. In some studies, it was more common in men, while in others it was reported to be significantly higher in the female gender.^13,22,23,26^ Colitis-related extraintestinal manifestations were more frequent in the colonic location of CD. This phenomenon probably has its explanation in the loss of the function of the intestine as a barrier, which occurs in inflammatory intestinal conditions and, in consequence, allows the flow of certain substances into systemic circulation mainly bacterial and food components at, predominantly, the colonic level, which would manage to reach the systemic circulation and activate the immune system, thus unleashing extraintestinal manifestations. Predominance in women, as seen in many other autoimmune conditions, may only be in relation to certain hormonal influences in the immune system. Triggers of the autoimmune responses in certain organs seem to be influenced by genetic factors. Concordance in EIM was present in 70% of parent–child pairs and 84% in sibling pairs. Associations of EIM in IBD with major histocompatibility complex loci have been demonstrated.^13,22^ In our study, we showed that skin findings were higher in patients with colon involvement and in female patients. In addition, it was shown that oral aphthae developed more frequently in patients with positive family history and in women. In addition, while there are parallels between some EIM findings and disease activity in studies, some are still unclear. While skin findings such as pyoderma gangrenosum have not been clarified in general, it has been reported that there is a relationship between other skin findings and oral stomatitis and disease activation.^10,20^ In our study, we showed that stomatitis and skin findings were higher in patients using corticosteroids, antibiotics, and anti-TNF drugs. In this context, closer follow-up of patients with mucocutaneous findings in terms of Crohn’s disease activation will contribute to the initiation of optimum treatment in a timely manner.

Perianal lesions such as erythema, ulcer, fissure, fistula, and abscess are common skin findings in CD patients but are not considered as an EIM.^24,27^ However, it has been shown that there are many common pathways such as intestinal pathology secondary to granulomatosis inflammation and neutrophil-related autoimmune inflammation as a result of the abnormal neutrophil function.^27,28^ In this context, Roth et al.^22^ reported a significant relationship between perianal abscess and the development of mucocutaneous findings. Similarly, in our study, there was a significant relationship between perianal disease and the development of skin findings.

Mucosal T cells play an important role in the continuation of intestinal homeostasis by providing a balance between mucosal epithelium and intestinal bacteria and host immune response. EIMs are known to occur due to lymphocyte-mediated destructive processes as a result of immune dysregulation.^29^ Mucosal T cells are thought to abnormally migrate from the intestines to the mucocutaneous space and cause damage as a result of exposure to antigens.^30^ In addition to many pathological pathways that play a role in skin involvement, the Th17 pathway and IL-17 expression have an important role, along with Th1 and Th2 lymphocytes. Th17 has been shown to play an important role in many inflammatory and autoimmune diseases, especially atopic dermatitis, psoriasis, Behçet’s disease, and systemic lupus erythematosus.^31^ In recent studies, IL-17 and IL-23 produced by Th17 are shown to contribute to the pathogenesis of IBD. It is thought that excessive IL-23 synthesis plays a role in the development of treatment-resistant skin findings.^32^ In addition, as the duration of the disease increases, it can be said that the development of other EIMs increases as a result of the increase in the exposure of neutrophil and lymphocyte-related autoimmune inflammation.

Although our study is important in terms of determining risk factors associated with the development of mucocutaneous findings in CD patients, there are some limitations. It may not reflect the whole Turkish population, since data is received only from a single tertiary center where complicated cases may cause a referral bias. Secondly, it should be noted that some findings may be overlooked due to the retrospective setting of the study. Our analyses for the relationship between the development of mucocutaneous findings and duration of disease, large sample size, long-term follow-up, and the evaluation of independent predictive risk factors affecting the development of mucocutaneous findings in CD patients are the strengths of our study.

In conclusion, the cumulative probability of skin involvement and oral aphthous ulcer development was found to be substantial in our study. We also showed that female gender, family history, colon involvement, and perianal disease play an important role in the development of mucocutaneous findings. It is suspected that there are common pathological pathways due to the existence of mucocutaneous findings before and after the diagnosis of CD. The immunological and clinical connections between these diseases and CD have not been fully elucidated. However, identifying specific relationships of immune-mediated diseases will help better understand the pathogenesis of CD and associated mucocutaneous findings to facilitate better treatment strategies.

## Figures and Tables

**Table 1.** The Relationship Between Skin Manifestations and Risk Factors in Patients with Crohn’s Disease

Risk Factors	Crohn’s Disease With Skin Manifestations N = 31 (9.2%)	Crohn’s Disease Without Skin Manifestations N = 305 (90.8%)	Total N = 336 (100%)	*P*
Age of diagnosis				.954
16 years and under	2 (6.4)	17 (5.6)	19 (5.7)	
Between 17 and 40 years	23 (74.2)	223 (73.1)	246 (73.2)	
40 years and above	6 (19.4)	65 (21.3)	71 (21.1)	
Sex				**.002 **
Female	22 (71)	128 (42)	150 (44.6)	
Male	9 (29)	177 (58)	186 (55.4)	
Age of disease				.417
10 years under	20 (64.5)	218 (71.5)	238 (70.8)	
10 years and above	11 (35.5)	87 (28.5)	98 (29.2)	
Involvement location (in the beginning)				**<.001**
Terminal ileum	6 (19.4)	115 (37.7)	121 (36)	
Colon	13 (41.9)	37 (12.1)	50 (14.9)	
Ileocolonic	10 (32.3)	143 (46.9)	153 (45.5)	
Upper GI tract	2 (6.5)	10 (3.3)	12 (3.6)	
Involvement location (end)				**.032**
Terminal ileum	6 (19.4)	106 (34.8)	112 (33.3)	
Colon	8 (25.8)	31 (10.2)	39 (11.6)	
Ileocolonic	15 (48.4)	158 (51.8)	173 (51.5)	
Upper GI tract	2 (6.5)	10 (3.3)	12 (3.6)	
Disease type				.370
Inflammatory	15 (48.4)	140 (45.9)	155 (46.1)	
Stenosis	3 (9.7)	60 (19.7)	63 (18.8)	
Penetrating	13 (41.9)	105 (34.4)	118 (35.1)	
Family history				.565
None	28 (90.3)	284 (93.1)	312 (92.9)	
Yes	3 (9.7)	21 (6.9)	24 (7.1)	
Perianal disease				**.009**
None	14 (45.2)	209 (68.5)	223 (66.4)	
Yes	17 (54.8)	96 (31.5)	113 (33.6)	
Surgical history				.886
None	18 (58.1)	173 (56.7)	191 (56.8)	
Yes	13 (41.9)	132 (43.3)	145 (43.2)	
Smoking				.459
Non-smoker	17 (54.8)	146 (47.9)	163 (48.5)	
Smoker	14 (45.2)	159 (52.1)	173 (51.5)	
Oral involvement				**<.001**
None	9 (29.0)	218 (71.5)	227 (67.6)	
Yes	22 (71.0)	87 (28.5)	109 (32.4)	

*P* < .05 value was considered statistically significant.

GI, gastrointestinal.

**Table 2.** The Relationship Between Skin Involvement and Medication in Crohn’s Patients

Medication	Crohn disease with skin manifestations N:31 %9.2	Crohn disease without skin manifestations N:305 %90.8	Total N:336 %100	P
Steroid				**.006**
* None *	1 (3.2)	77 (25.2)	78 (23.2)	
* Yes *	30 (96.8)	228 (74.8)	258 (76.8)	
Immunemodulator				.186
* None *	3 (9.7)	59 (19.3)	62 (18.5)	
* Yes *	28 (90.3)	246 (80.7)	274 (81.5)	
Antibiotic				**.013**
* None *	5 (16.1)	118 (38.7)	123 (36.6)	
* Yes *	26 (83.9)	187 (61.3)	213 (63.4)	
Anti-TNF				**.025**
* None *	16 (51.6)	217 (71.1)	233 (69.3)	
* Yes *	15 (48.4)	88 (28.9)	103 (30.7)	

*P* < .05 value was considered statistically significant.

**Table 3.** The Relationship Between Oral Involvement and Risk Factors in Crohn’s Patients

Risk factors	Crohn disease with oral involvement N:109 %32.4	Crohn disease without oral involvement N:227 %67.6	Total N:336 %100	P
Age of diagnosis				.336
* 16 years and under *	5 (4.6)	14 (6.2)	19 (5.7)	
* Between 17-40 years *	76 (69.7)	170 (74.9)	246 (73.2)	
* 40 years and above *	28 (25.7)	43 (18.9)	71 (21.1)	
Sex				**.015**
* Female *	59 (54.1)	91 (40.1)	150 (44.6)	
* Male *	50 (45.9)	136 (59.9)	186 (55.4)	
Age of Disease				.065
* 10 years under *	70 (64.2)	168 (74)	238 (70.8)	
* 10 years and above *	39 (35.8)	59 (26)	98 (29.2)	
Involvement location (in the beginning)				**.038**
* Terminal ileum *	35 (32.1)	86 (37.9)	121 (36.0)	
* Colon *	25 (22.9)	25 (11.0)	50 (14.9)	
* Ileocolonic *	46 (42.2)	107 (47.1)	153 (45.5)	
* Upper GI tract *	3 (2.8)	9 (4.0)	12 (3.6)	
Involvement location (end)				.136
* Terminal ileum *	35 (32.1)	77 (33.9)	112 (33.3)	
* Colon *	19 (17.4)	20 (8.8)	39 (11.6)	
* Ileocolonic *	52 (47.7)	121 (53.3)	173 (51.5)	
* Upper GI tract *	3 (2.8)	9 (4.0)	12 (3.6)	
Disease type				**.012**
* Inflammatory *	63 (57.8)	92 (40.5)	155 (46.1)	
* Stenosis *	16 (14.7)	47 (20.7)	63 (18.8)	
* Penetrating *	30 (27.5)	88 (38.8)	118 (35.1)	
Family history				**<.001**
* None *	90 (82.6)	222 (97.8)	312 (92.9)	
* Yes *	19(17.4)	5(2.2)	24 (7.1)	
Perianal disease				.933
* None *	72 (66.1)	151 (66.5)	223 (66.4)	
* Yes *	37 (33.9)	76 (33.5)	113 (33.6)	
Surgical history				**.033**
* None *	71 (65.1)	120 (52.9)	191 (56.8)	
* Yes *	38 (34.9)	107 (47.1)	145 (43.2)	
Smoking				.336
* Non-smoker *	57 (52.3)	106 (46.7)	163 (48.5)	
* Smoker *	52 (47.7)	121 (53.3)	173 (51.5)	

P < .05 value was considered statistically significant.

**Table 4.** The Relationship Between Oral Involvement and Medication in Crohn’s Patients

Medication	Crohn disease with oral involvement N:109 %32.4	Crohn disease without oral involvement N:227 %67.6	Total N:336 %100	P
Steroid				.097
* None *	19 (17.4)	58 (25.6)	77 (22.9)	
* Yes *	90 (82.6)	169 (74.4)	259 (77.1)	
Immunemodulator				.790
* None *	21 (19.3)	41 (18.1)	62 (18.5)	
* Yes *	88 (80.7)	186 (81.9)	274 (81.5)	
Antibiotic				.645
* None *	38 (34.9)	85 (37.4)	123 (36.6)	
* Yes *	71 (65.1)	142 (62.6)	213 863.4)	
Anti-TNF				.917
* None *	76 (69.7)	157 (69.2)	233 (69.3)	
* Yes *	33 830.3)	70 (30.8)	103 (30.7)	

**Table 5.** Multivariate Cox Regression Analysis of Independent Risk Factors Associated with Mucocutaneous Manifestations Development

Dependent variable	Independent variable	HR	Cl %95	*P*
Skin involvement				
	Sex	3.280	1.507-7.138	.003
	Steroid use	7.881	1.072-57.966	.043
Oral involvement				
	Inflammatory type	1.776	1.207-2.614	.004
	Family history	3.593	2.176-5.934	<.001
